# “Reconsidering CYP3A4/5 Genotyping for Ticagrelor Safety: Critical Appraisal of a Narrow Genetic Framework”

**DOI:** 10.1002/clc.70251

**Published:** 2025-12-29

**Authors:** Ibadullah Tahir, Hunain Shahbaz

**Affiliations:** ^1^ Ayub Medical College Abbottabad Pakistan

Dear Editor,

While we found the article by Zhang and collaborators to be very interesting, numerous methodological limitations impair the reliability and clinical usefulness of the results and conclusions. Of particular concern is the limitation of the genetic scope to two SNPs, CYP3A4 rs2242480 and CYP3A5 rs776746. This restricts the research to only acting on two SNPs that were indicated as being involved in the pharmacokinetics and/or pharmacodynamics of ticagrelor and other agents that produce adverse effects.

Prior studies have firmly established the roles of SLCO1B1, ABCB1, UGT2B7, and P2Y12 allele variations in influencing ticagrelor bioavailability and dyspnea risk [1, 2, 3]. If the current study does not account for these genes, it may result in a large‐scale shortage of genetic information to assess and incorrectly attribute the effects of other genes and environmental variables to just one SNP. It is also very disconcerting that key clinical outcomes are clearly underpowered. Low rates of ticagrelor‐associated bleeding/revascularization events were recorded; a prior pharmacogenomic study indicated that to detect the genetic modality of rare events, one must conduct studies with large, multi‐institutional cohorts [4].

The evidence of significantly low statistical power regarding major clinical outcomes evaluated is equally alarming. The frequency of ticagrelor‐related occurrence of bleeding and revascularization is low, and previous pharmacogenomic studies identified that large, multicenter studies have the necessary statistical power to detect the effects of genetic variations on these rare clinical outcomes [4]. The small number of occurrences reported by Zhang et al. (e.g., 13 bleeding events) severely limited statistical power, resulting in an increased possibility of producing false negative results, thus making their conclusion of “no association” highly questionable.

The results of this study show a higher training accuracy but a significantly lower testing accuracy than what was found in other studies, indicating an unstable model. The authors, however, interpret this result as having biological meaning; a more cautious approach would take into account the risk of overfitting that GMDR has when using a large number of training sets [5]. In addition to the lack of confounding variables for the evaluation of dyspnea, there are multiple reasons why patients taking ticagrelor may experience dyspnea due to conditions other than the drug itself, for example, baseline pulmonary condition, vagal sensitivity, renal function, and other prescriptive medications, including β‐blockers and ACE inhibitors [3, 4, 5, 6].

Studies have shown that renal dysfunction and platelet reactivity are independent predictors of the severity of dyspnea [3, 4, 5, 6, 7]. The paper by Zhang et al. excluded several cardiopulmonary disorders; however, they did not rectify for the residual variability of the lung function or any concomitant therapy that would likely impact the time of symptom development. Without appropriate adjustment, it is likely that there was some degree of confounding related to the genetic association reported.

In conclusion, the authors have been overly optimistic in regard to the clinical application of genotyping. Currently, there is no professional association (ACC, AHA, ESC, or CPIC) that recommends performing pharmacogenetic testing for ticagrelor. In contrast to clopidogrel, which requires dose adjustment based on genotype, experts agree that the efficacy of ticagrelor is not significantly affected by the common CYP polymorphisms [1, 2]. At this time, until verified through larger‐scale prospective studies, we consider the idea of using rs776746 as part of an overall ACS treatment plan to be inappropriately premature and potentially misleading to healthcare providers.

While Zhang et al.'s paper adds to the current body of knowledge regarding the tolerability of ticagrelor, they did so with underpowered studies and with unknown confounding variables, a small amount of genetic data, and by overinterpreting exploratory results. Therefore, adequate multi‐locus powered studies with independent validation of the data must be conducted before genotyping of ticagrelor becomes part of clinical practice.

## Author Contributions

All authors have read and approved the final version of the manuscript.

## Funding

The authors received no specific funding for this work.

## Ethics Statement

The authors have nothing to report.

## Consent

The authors have nothing to report.

## Conflicts of Interest

The authors declare no conflicts of interest.

## Data Availability

The authors have nothing to report.
